# Impact of comorbidities on treatment management and prognosis in patients with anaplastic thyroid cancer (ATC)

**DOI:** 10.1007/s00432-025-06403-7

**Published:** 2025-12-23

**Authors:** Teresa Augustin, Dmytro Oliinyk, Marlen Haderlein, Charlotte Frei, Julia Jacob, Daniel Medenwald, Maike Trommer, Matthias Mäurer, Sonia Drozdz, Alexander Rühle, Anca-Ligia Grosu, Nils Henrik Nicolay, Maria Waltenberger, Stephanie E. Combs, Anastassia Löser, Michael Oertel, Hans Theodor Eich, Stefan Janssen, Josefine Rauch, Ralph Gurtner, Robert Renollet, Christine Spitzweg, Dirk Vordermark, Claus Belka, Lukas Käsmann

**Affiliations:** 1 Department of Radiation Oncology, University Hospital, LMU Munich, 81377 Munich, Germany; 2 Department of Plastic, Hand and Reconstructive Surgery, University Hospital Regensburg, Franz-Josef-Strauß Allee 11, 93053 Regensburg, Germany; 3 Department of Radiation Oncology, Universitätsklinikum Erlangen, Friedrich-Alexander-Universität Erlangen-Nürnberg, Erlangen, Germany; 4 Comprehensive Cancer Center Erlangen-EMN (CCC ER-EMN), Universitätsklinikum Erlangen, Friedrich-Alexander-Universität Erlangen-Nürnberg, Erlangen, Germany; 5 Department of Radiation Oncology, University Hospital Halle (Saale), Ernst-Grube- Str. 40, 06120 Halle (Saale), Germany; 6 Institute of Medical Epidemiology, Biometry, and Informatics, Martin Luther University Halle-Wittenberg, Magdeburger Straße 8, 06112 Halle (Saale), Germany; 7 Department of Radiation Oncology, Faculty of Medicine, University Hospital Bonn, Bonn, Germany; 8 Department I of Internal Medicine, Center for Integrated Oncology CIO Aachen Bonn Cologne Duesseldorf, University Hospital of Cologne, Cologne, Germany; 9 Department of Radiation Oncology, Olivia Newton-John Cancer Wellness and Research Centre, Austin Health, Melbourne, Australia; 10 Department for Radiotherapy and Radiation Oncology, University Hospital Jena, Friedrich-Schiller-University, Am Klinikum 1, 07747 Jena, Germany; 11 Clinician Scientist Program “OrganAge”, Jena University Hospital, 07747 Jena, Germany; 12 Department of Radiation Oncology, Medical Center, Faculty of Medicine, University of Freiburg, 79106 Freiburg, Germany; 13 Department of Radiotherapy and Radiation Oncology, University Hospital Leipzig, 04103 Leipzig, Germany; 14 Department of Radiation Oncology, Klinikum rechts der Isar, TUM School of Medicine and Health, Technical University of Munich, Munich, Germany; 15 Department of Radiation Oncology, Hospital of Bolzano (SABES-ASDAA), Bolzano, Italy; 16 Partner-site Munich and German Cancer Research Center (DKFZ), Heidelberg, Germany; 17 Institute of Radiation Medicine, Helmholtz Zentrum München, Neuherberg, Germany; 18 Department of Radiotherapy, University Medical Center Schleswig-Holstein, Campus Lübeck, Ratzeburger Allee 160, 23538 Lübeck, Germany; 19 Department of Radiotherapy and Radiation Oncology, University Medical Center Hamburg-Eppendorf, Outpatient Center of the UKE GmbH, Hamburg, Germany; 20 Department of Radiation Oncology, University Hospital Muenster, Muenster, Germany; 21 Private Practice of Radiation Oncology, Hannover, Germany; 22 Department of Internal Medicine IV, University Hospital, LMU Munich, Munich, Germany; 23 Adjunct Academic Appointment, Division of Endocrinology, Diabetes, Metabolism and Nutrition, Mayo Clinic Rochester, Rochester, MN USA; 24 German Cancer Consortium (DKTK), Partner Site Munich, Munich, Germany; 25 Bavarian Cancer Research Center (BZKF), Munich, Germany; 26 Department of Radiation Oncology, Klinikum Landshut, Robert-Koch-Str. 1, 84034 Landshut, Germany

**Keywords:** ATC, Prognosis, Comorbidity, Treatment allocation, Real-word data

## Abstract

To evaluate the impact of comorbidities on treatment allocation and prognosis in anaplastic thyroid cancer, 137 patients from 10 German tertiary cancer centers treated with radiotherapy between 2001 and 2020 were analyzed. Four validated comorbidity scores were applied to assess comorbidity burden. The primary objective was to identify prognostic factors for the survival rate at 6 months after radiotherapy and discriminate the comorbidity scores using concordance statistics, ROC curve net reclassification index, and integrated discrimination improvement for 6-month survival. The median overall survival (OS) of the entire cohort was 4 months (95% CI = 2.72–5.28). The 6-, 12- and 24-months survival rates were 42.1%, 29.0% and 15.0%, respectively. In the univariate analysis, Karnofsky Performance Score (KPS) (> 70%, *p* < 0.001), UICC stage (*p* < 0.001), treatment modality (*p* < 0.001), intention of treatment (*p* < 0.001) as well as lower scores in the conventional Charlson Comorbidity Index (cCCI, *p* < 0.001), the updated Charlson Comorbidity Index (uCCI, *p* < 0.001) were associated with improved OS. KPS (> 70%, *p* = 0.06) and type of therapy (*p* = 0.087) showed a trend in multivariate analysis. Higher comorbidity burden (cCCI and uCCI) was associated with less intensive treatment and lower cumulative radiation doses in univariable analyses. However, after adjustment for age and metastatic status, none of the comorbidity indices remained independently associated with the use of multimodal therapy or the prescribed EQD2 dose (*p* > 0.05). Age, but not metastatic status, was linked to a reduced likelihood of receiving multimodal treatment. In contrast, KPS emerged as the only independent predictor of higher EQD2 dose levels in the multivariable models.

**Supplementary Information:**

The online version contains supplementary material available at 10.1007/s00432-025-06403-7.

## Introduction

Anaplastic Thyroid Cancer (ATC) is with 1–2% the rarest subtype of all thyroid carcinomas. However, it is responsible for about 50% of all thyroid-cancer-associated deaths (Salehian et al. 2019; Sugitani et al. 2018; Bible et al. 2021).

The median overall survival (OS) in ATC without targetable mutations ranges between 3 and 6 months with a 1-year survival rate of 10–20% (Sugitani et al. 2018; Onoda et al. 2020; Pezzi et al. 2017; Filetti et al. 2019). Treatment allocation depends on certain patient- and tumor-related characteristics with prognostic significance. Occurrence of metastases, age at initial diagnosis, type of therapy and radiation doses have already been investigated and identified as prognostic factors for OS (Pezzi et al. 2017; Filetti et al. 2019; Haymart et al. 2013; Corrigan et al. 2019; Lee et al. 2018; Sun et al. 2013; Fan et al. 2019; Glaser et al. 2016; Wendler et al. 2016). Identifying prognostic factors contributes to further optimization and personalization of conventional therapies like surgery, radiation and systemic therapy.

Elderly people (≥ 65 years) represent an important and heterogenous subgroup of ATC patients, often presenting with a variety of comorbidities, multi-medication and reduced organ function that complicate therapeutic decision-making and may impact treatment intensity (Pezzi et al. 2017; Glaser et al. 2016; Gui et al. 2020; Li et al. 2019). To provide more precise and individualized treatment strategies, comorbidities especially in older patients, must be considered for treatment planning and further investigated as a factor with potential impact on the outcome of ATC patients.

In other tumor entities like lung cancer (Kaesmann et al. 2016; Asmis et al. 2008), colorectal cancer (Ouellette et al. 2004), head and neck cancer (Sanabria et al. 2007; Wang et al. 2016; Göllnitz et al. 2016) as well as differentiated thyroid cancer (Lee et al. 2019) comorbidities scores have been proven to be an independent factor predicting OS. However, there is a lack of similar data for patients with ATC.

In the present study, comorbidity burden was investigated using the Charlson Comorbidity Score (CCI), two of its modifications and the Simplified Comorbidity Score (SCS) regarding their potential impact on treatment management and survival in ATC patients.

## Patients and methods

### Patient cohort

In this real-world, multicenter study (DRKS00032180), we retrospectively included all consecutively treated patients with anaplastic thyroid cancer (ATC) who received radiotherapy between January 2001 and January 2020 at 10 tertiary cancer centers in Germany. The multicenter cohort initially comprised data from 158 patients. After excluding patients with incomplete information regarding comorbidities and/or patient characteristics (*n* = 21), the final study population consisted of 137 (86.7%) patients.

### Data acquisition and classification

Data were analyzed according to patient- and treatment-related characteristics like age, gender, Karnofsky performance status (KPS), comorbidities, Union for International Cancer Control Tumor–Node–Metastasis (UICC TNM) classification (8th edition), intention of treatment, type of therapy, performance of surgery and applied chemotherapy regimens. Only patients with histologically confirmed ATC, staged according to the UICC TNM classification and fully available information on comorbid diseases were included. OS was defined as time between the last day of radiotherapy and death. Patients still alive or lost to follow-up were censored at the time of last visit. Multimodal treatment was defined based on earlier reports such as trimodal therapy containing surgical resection and postoperative chemoradiotherapy (CRT) (Fan et al. 2019; Haddad et al. 2022). The primary endpoint of the study was the 6-months survival rate and additional endpoints were 12- and 24-months survival rates.

### Comorbidity assessment

Comorbidities were assessed by referring to a definition, which indicates comorbid diseases as two or more medical conditions existing simultaneously regardless of their causal relationship (Charlson et al. 1987). One of the most frequently used scores is the conventional Charlson Comorbidity Index (cCCI), which assigns 1,2,3 or 6 points to 19 comorbidities with a maximum comorbidity score of 33 points (Charlson et al. 1987). We did not record metastatic solid tumors and anaplastic thyroid cancer as comorbid conditions. The updated version of the Charlson Comorbidity Index (uCCI) assigns 0,2,4 or 6 points to 17 different comorbidities with a maximum score of 24 points (see Supplementary Table SI) (Deyo et al. 1992). Since age and occurrence of comorbidities are closely related, a combination of both covariates within the age-adapted Charlson Comorbidity Index (aaCCI) has shown to be a highly significant predictor of prognosis (Charlson et al. 1994). It was originally designed for small studies and is primarily used in oncology (Charlson et al. 2022). For the aaCCI, 1 point for every 10 years over the age of 50 is added to final cCCI (Robbins et al. 2013). Finally, the Simplified Comorbidity Score (SCS) was calculated (Colinet et al. 2005). The SCS assigns 1, 4, 5 or 7 points to 7 different comorbidities (see Supplemetary Table SII).

### Statistical analyses

The survival analyses were performed using the R programming language (version 3.5.7). The analyses utilized the survival (version 3.5.7) and survminer (version 0.5.0) packages. We used the median value as the cutoff point for each comorbidity score to dichotomize patient cohort. Given the retrospective and hypothesis-generating nature of this real-world study, no formal sample size calculation or statistical power estimation was conducted in advance.

The concordance index was calculated using the survConcordance function of the package survival v3.5.7. The “roc-test” function of the package “pROC” v1.18.4 was used to calculate De Long´s test for AUC of the receiver operating characteristic curve (ROC) comparison. The plot of superimposed AUC of the ROC of the models used was generated by the “plotROC” function of the package “predictABEL” v1.2–4. Time-dependent ROC curve analysis was conducted using the timeROC function of the package timeROC v0.4. The Continuous Net Reclassification Improvement (NRI) and Integrated Discrimination Improvement (IDI) were calculated using the IDI.INF function of the package survIDINRI v1.1.2.

### Institutional review board (IRB) approval and patient consent

The study protocol was approved by the ethics committee of the Ludwig-Maximilians-University of Munich (Munich, Germany) (approval number: 19-885). Additionally, institutional review boards at each participating center approved both the data collection process and the sharing of data with the primary study center. Due to the retrospective nature of the study and the anonymized handling of patient data, individual patient consent was waived by the responsible ethics committees. All procedures adhered strictly to the ethical standards outlined in the Declaration of Helsinki.

## Results

### Patient characteristics

The entire cohort consisted of 137 patients with a median age of 68 years (range: 29–97 years). Of all patients, 79 (57.7%) were female and 58 (42.3%) were male. Thirty-seven (27.0%) patients had a KPS of ≤ 70%, 55 (40.1%) of > 70%. The vast majority of patients (*n* = 112, 81.7%) had T-stage 4, 23 (16.8%) T-stage 3, one (0.7%) had T-stage 2 and for one (0.7%) patient T-stage was unknown. Furthermore, 41 (29.9%) patients showed no nodal involvement (N-stage). At initial diagnosis, 6 (4.4%) patients showed a disease limited to the thyroid gland (UICC stage IVA), 52 (38.0%) showed extrathyroidal infiltrations (UICC stage IVB), and 75 (54.7%) patients already had distant metastasis (UICC stage IVC). Next generation sequencing (NGS) was performed in 30 (21.9%) patients and BRAF V600E mutation-testing was conducted in 29 (21.2%) patients. Amongst those tested, five (17.2%) patients were found to have a BRAF V600E mutation.

As for the scores within the four comorbidity indices, there were 75 (54.7%) patients with less than 2 points in the cCCI, 56 (40.9%) with 2 or more points (range: 0–6, median: 2 points). Regarding the uCCI, 76 (55.5%) patients had a score of < 2 and 61 (44.5%) patients of ≥ 2 points. The distribution of the groups within the aaCCI was as follows: 67 (51.1%) with less than 4 points, 64 (46.7%) with ≥ 4 points and unknown for 6 (4.4%) patients. For the SCS, 56 (40.9%) patients had less than 2 points, 56 (40.9%) had 6 or more points and for 25 (18.2%) patients the score was unknown (see Table 1).

**Table 1:** Patient- and treatment-related characteristics for the pooled cohort

Parameter	Number (%)
*Age, Years* < 70 ≥ 70	73 (53.3%) 64 (46.7%)
*Gender* Female Male	79 (57.7%) 58 (42.3%)
*KPS, %* ≤ 70 > 70 Unknown	37 (27.0%) 55 (40.1%) 45 (32.8%)
*T-Stage* 2 3 4 Unknown	1 (0.7%) 23 (16.8%) 112 (81.7%) 1 (0.7%)
*Nodal involvement* No Yes Uncertain	41 (29.9%) 50 (36.5%) 46 (33.6%)
*UICC Stage* IVA-B IVC Unknown	58 (42.3%) 75 (54.7%) 4 (2.9%)
*cCCI, points* < 2 ≥ 2 Unknown	75 (54.7%) 56 (40.9%) 6 (4.4%)
*uCCI, points* < 2 ≥ 2	76 (55.5%) 61 (44.5%)
*aaCCI, points* < 4 ≥ 4 Unknown	67 (51.1%) 64 (46.7%) 6 (4.4%)
*SCS, points* < 2 ≥ 2 Unknown	56 (40.9%) 56 (40.9%) 25 (18.2%)
*Type of therapy* Surgery + CRT Surgery + RT RT + conChT RT alone	50 (36.5%) 41 (29.9%) 23 (16.8%) 23 (16.8%)
*Surgery* Hemithyreoidectomy Subtotal Thyreoidectomy Total Thyreoidectomy Unknown	91 (66.4%) 21 (23.1%) 15 (16.5%) 35 (38.5%) 20 (22.0%)
*Radiation dose level in EQD2* < 40 Gy 40–60 Gy > 60 Gy	23 (16.8%) 66 (48.2%) 48 (35.0%)
*Concurrent Chemotherapy* Carboplatin/Paclitaxel Doxorubicin Cisplatin Other combinations	73 (53.3%) 24 (32.9%) 14 (19.2%) 17 (23.3%) 18 (24.7%)
*Curative Treatment Intention* Yes No	55 (40.1%) 82 (59.9%)

KPS, Karnofsky Performance Status; T-Stage, Tumor-Stage; UICC, Union for International Cancer Control; cCCI, conventional Charlson Comorbidity Score; uCCI, updated Charlson Comorbidity Score; aaCCI, age-adapted Charlson Comorbidity Score; SCS, Simplified Comorbidity Score; CRT, Chemoradiotherapy; RT, Radiotherapy; conCHT, concurrent Chemotherapy

### Treatment characteristics

Of the 137 patients, 23 (16.8%) received radiotherapy alone, 23 (16.8%) radiotherapy with concurrent chemotherapy, 41 (29.9%) had surgery followed by adjuvant radiotherapy, and 50 (36.5%) were treated with surgery followed by postoperative chemoradiation. Among the 91 (66.4%) patients who underwent surgery 21 (23.1%) had a hemi-, 15 (16.5%) a subtotal and 35 (38.5%) a total thyroidectomy, for 20 (22.0%) patients information on resection status was missing. Radiotherapy techniques included 3D-conformal radiotherapy (3D-RT, 35.3%), intensity-modulated radiotherapy (IMRT, 32.4%), and volumetric-modulated arc therapy (VMAT, 24.5%). A smaller subset of patients was treated with other techniques, including 2D/opposing-field approaches and helical tomotherapy (combined 7.8%). Concurrent chemotherapy was performed in 73 (53.3%) patients, of which 24 (32.9%) received a combination of Carboplatin/Paclitaxel, 14 (19.2%) a single-agent therapy with Doxorubicin, 17 (23.3%) one with Cisplatin and 18 (24.7%) patients underwent other chemotherapeutic regimens. Eighty-two (59.9%) patients were treated with palliative and 55 (40.1%) with curative intent (see Table 1). Patients with palliative treatment intention were irradiated with a median EQD2 (α/β = 10 Gy) of 48.75 Gy (Interquartile range (IQR), 41.01–56.80), those with curative intent with a median EQD2 of 64.0 Gy (IQR, 60.0–67.1).

### Outcome

For the pooled cohort the median OS was 4 months (95% Confidence Interval (95% CI) = 2.72–5.28). The 6-, 12- and 24-months survival rates were 42.1%, 29.0% and 15.0%, respectively.

**Fig. 1:**
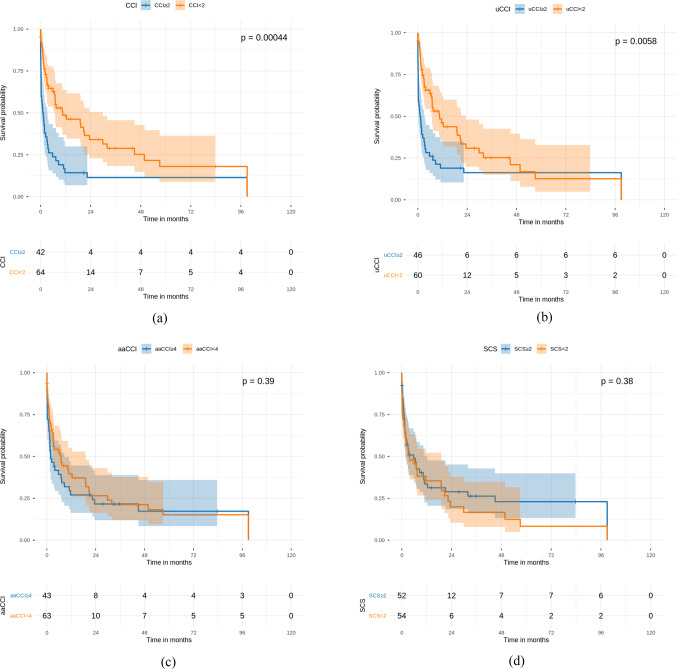
Kaplan-Meier curves and survival time rates for cCCI (**a**), uCCI (**b**), aaCCIaa (**c**) and SCS (**d**). cCCI, conventional Charlson Comorbidity Score; uCCI, updated Charlson Comorbidity Score; aaCCI, age-adapted Charlson Comorbidity Score; SCS, Simplified Comorbidity Score.

In the univariate analysis, KPS (> 70%), UICC stage, type of therapy, intention of treatment as well as the cCCI (< 2 points), uCCI (< 2 points) were associated with improved OS (see Table 2; Fig. 1a–d).

**Table 2:** Uni- and multivariate analysis of overall survival in the pooled cohort

				Univariate analysis		Multivariate analysis	
Parameter	6-month survival	12-month survival	24-month survival	*p* value	HR [95% CI]	*p* value	HR [95% CI]
*Age* < 70 ≥ 70	45.2% 38.5%	26.7% 29.3%	12.7% 17.8%	0.68	0.92 [0.62–1.36]		
*Gender* Female Male	43.4% 40.7%	30.5% 24.8%	15.0% 14.9%	0.85	0.96 [0.65–1.43]		
*KPS* ≤ 70 > 70	10.4% 60.2%	5.2% 34.9%	0.0% 18.2%	**< 0.001**	**0.26 [0.15–0.45]**	0.06	0.48 [0.22–1.04]
*UICC stage* IVA-B IVC	60.8% 26.8%	46.3% 15.5%	29.2% 8.6%	**< 0.001**	**0.40 [0.26–0.60]**	0.19	0.52 [0.19–1.39]
*Type of therapy* Surgery + CRT Surgery + RT RT + conChT RT alone	55.9% 48.9% 19.6% 22.7%	29.6% 42.1% 19.6% 8.5%	14.8% 27.3% 0.0% 8.5%	**< 0.001**	**0.67 [0.56–0.81]**	0.087	0.76 [0.55–1.04]
*Curative treatment intention* Yes No	63.8% 28.4%	45.9% 15.5%	25.2% 7.0%	**< 0.001**	**0.39 [0.25–0.60]**	0.841	0.92 [0.41–2.08]
*cCCI* < 2 ≥ 2	57.3% 24.4%	42.8% 14.6%	25.3% 12.2%	**< 0.001**	**0.45 [0.30–0.70]**	0.605	0.74 [0.33–4.25]
*uCCI* < 2 ≥ 2	61.4% 22.2%	46.2% 12.5%	25.9% 12.5%	**< 0.001**	**0.42** **[0.27–0.66]**	0.968	0.97 [0.26–3.70]
*aaCCI* < 4 ≥ 4	40.0% 46.9%	27.5% 33.7%	25.0% 17.1%	0.39	0.93 [0.60–1.43]		
*SCS* < 2 ≥ 2	46.0% 42.2%	28.9% 34.2%	22.0% 17.8%	0.38	1.06 [0.69–1.63]		

Bold figures indicate statistical significance (*p* < 0.05)

HR, Hazard Ratio; CI, Confidence Interval; KPS, Karnofsky Performance Status; UICC, Union for International Cancer Control; CRT, Chemoradiotherapy; RT, Radiotherapy; conCHT, concurrent Chemotherapy; cCCI, conventional Charlson Comorbidity Score; uCCI, updated Charlson Comorbidity Score; aaCCI, age-adapted Charlson Comorbidity Score; SCS, Simplified Comorbidity Score

KPS (Hazard ratio (HR) = 0.48, 95% CI = 0.22–1.04, *p* = 0.06) and type of therapy (HR = 0.76, 95% CI = 0.55–1.04, *p* = 0.087) showed a trend in the multivariate analysis. All factors included in the multivariate analysis did not achieve significance (see Table 2).

**Fig. 2:**
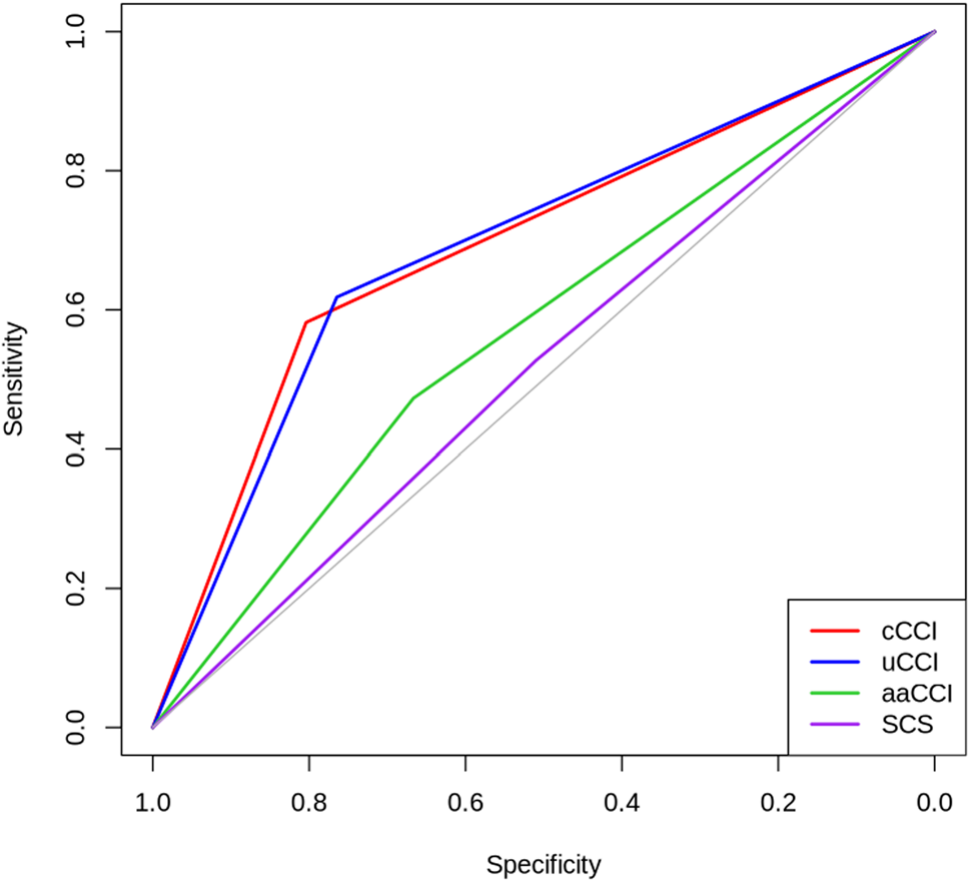
ROC curve for all comorbidity scores. cCCI, conventional Charlson Comorbidity Score; uCCI, updated Charlson Comorbidity Score; aaCCI, age-adapted Charlson Comorbidity Score; SCS, Simplified Comorbidity Score

We first used a binomial logistic regression model to analyze the relationship between the binary dependent variable OS at 6 months and the risk groups calculated according to the comorbidity scores. The logistic regression model using cCCI performed slightly better than the uCCI, with an odds ratio of 0.18 (95% CI 0.07–0.42), *p* < 0.01) compared to 0.19 (95% CI 0.08–0.44), *p* < 0.01). The area under the ROC curve was marginal higher for the cCCI compared to uCCI (AUC of the ROC 0.693 vs. 0.691; DeLong’s test *p* = 0.95). (see Fig. 2).

The optimal groups for binary 6-month OS prediction were calculated for each regression model. Patients with lower cCCI or uCCI scores had an increased probability for survival at 6 months. The aaCCI and SCS showed lower discriminative ability with AUCs of 0.64 and 0.54 respectively, suggesting that they may be less suitable for predicting 6-month survival in our cohort.

The cCCI risk group model exhibited a concordance index (C-index) of 0.37 (95% CI 0.31–0.43, *p* < 0.01), indicating limited discriminatory power of the model in ranking individuals according to their 6-month OS rate (see Table 3).

**Table 3:** Comorbidity scores and corresponding area under the ROC curve, concordance index and odds ratio for death at 6 months

Comorbidity score	Area under the ROC curve (95% CI) for death at 6 months	Concordance index (95% CI *p* value)	Odds ratio (95% CI *p* value) for death at 6 months
cCCI	0.693 (95% CI 0.61–0.78)	0.37 (95% CI 0.31–0.43; *p* < 0.01)	0.18 (95% CI 0.07–0.42), *p* < 0.01
uCCI	0.691 (95% CI 0.60–0.78)	0.37 (95% CI 0.31–0.43; *p* < 0.01)	0.19 (95% CI 0.08–0.44), *p* < 0.01
aaCCI	0.43 (95% CI 0.34–0.52)	0.46 (95% CI 0.39–0.52; *p* = 0.16)	0.56 (95% CI 0.25–1.22), *p* = 0.15
SCS	0.52 (95% CI 0.42–0.61)	0.52 (95% CI 0.46–0.58; *p* = 0.56)	1.16 (95% CI 0.54–2.49), *p* = 0.70

CI, Confidence Interval; cCCI, conventional Charlson Comorbidity Score; uCCI, updated Charlson Comorbidity Score; aaCCI, age-adapted Charlson Comorbidity Score; SCS, Simplified Comorbidity Score

The uCCI and cCCI model have a highly similar concordance index of 0.37 (95% CI 0.31–0.43; *p* < 0.01). Although the concordance indices are relatively low, they still provide predictive ability for the models. The aaCCI and SCS models showed higher concordance indices of 0.46 (95% CI 0.39–0.52; *p* = 0.16) and 0.52 (95% CI 0.46–0.58; *p* = 0.56), respectively.

The NRI quantifies the improvement in risk prediction of one model compared to another model. It provides an assessment of the extent to which the new model improves risk stratification. We calculated the NRI on the logistic regression models for all score comparisons. Compared with the cCCI-based model, neither the uCCI-based model nor the models using aaCCI or SCS showed an improvement in risk stratification. In fact, the overall NRI values for aaCCI and SCS versus cCCI were significantly negative, indicating that replacing cCCI with these alternative scores leads to worse reclassification of patients with respect to 6‑month mortality risk (see Table 4).

**Table 4:** Model comparison and corresponding NRI and IDI

Model comparison	NRI (95% CI *p *value)	IDI (95% CI *p *value)
cCCI vs. uCCI	−0.04 (95% CI −0.21 − 0.12; *p* = 0.59)	−0.01 (95% CI −0.04 − 0.03; *p* = 0.73)
aaCCI vs. cCCI	−0.77 (95% CI −1.11 − 0.43; *p* < 0.001)	−0.14 (95% CI −0.19 − 0.08; *p* < 0.001)
SCS vs. cCCI	−0.77 (95% CI −1.11 − 0.43; *p* < 0.001)	−0.15 (95% CI −0.22 − 0.08; *p* < 0.001)

NRI; Net Reclassification Improvement; IDI, Integrated Discrimination Improvement; CI, Confidential Interval; cCCI, conventional Charlson Comorbidity Score; uCCI, updated Charlson Comorbidity Score; aaCCI, age-adapted Charlson Comorbidity Score; SCS, Simplified Comorbidity Score

The uCCI model showed no meaningful net gain in reclassification compared with cCCI, with an NRI that was close to zero and not statistically significant.

The IDI comparing the logistic regression models evaluates the change in the difference between average predicted risks for those who experienced the event and those who did not. In line with the NRI findings, the IDI did not support a meaningful improvement of uCCI, aaCCI, or SCS over cCCI. The IDI values for models based on aaCCI and SCS versus cCCI were significantly negative, indicating a reduced separation between predicted risks in survivors and non-survivors when cCCI was replaced by these scores. For uCCI versus cCCI, the IDI was small and not statistically significant, again suggesting no relevant gain in overall discrimination (see Table 4).

### Comorbidities and treatment management

Surgery was found to be more often applied in patients with lower comorbidity scores. Both the cCCI < 2 and the uCCI < 2 showed a statistically significant association with the applied treatment regimen (cCCI: χ^2^(3) = 11.96, *p* = 0.008, Cramer’s V = 0.34; uCCI: χ^2^(3) = 13.69, *p* = 0.003, Cramer’s V = 0.36), indicating that patients with lower comorbidity scores were more likely to receive surgery-based multimodal treatment. In contrast, neither the aaCCI (cut-off 4 points; χ²(3) = 3.50, *p* = 0.32, Cramer’s V = 0.18) nor the SCS (cut-off 2 points; χ^2^(3) = 4.91, *p* = 0.18, Cramer’s V = 0.22) demonstrated a statistically significant correlation with treatment type (see Table 5).

**Table 5:** Correlation between comorbidity score and applied treatment regimen

	Type of therapy (radiotherapy vs. chemoradiotherapy vs. surgery with adjuvant radiotherapy vs. surgery with adjuvant chemoradiotherapy)		
	χ^2^	*p* value	Cramer’s V
cCCI (< 2 vs. ≥2)	11.96 (3)	**0.008**	0.34
uCCI (< 2 vs. ≥2)	13.69 (3)	**0.003**	0.36
aaCCI (< 4 vs. ≥4)	3.50 (3)	0.32	0.18
SCS (< 2 vs. ≥2)	4.91 (3)	0.18	0.22

Bold figures indicate statistical significance (*p* < 0.05)

cCCI, conventional Charlson Comorbidity Score; uCCI, updated Charlson Comorbidity Score; aaCCI, age-adapted Charlson Comorbidity Score; SCS, Simplified Comorbidity Score

In a multivariable logistic regression model adjusted for age and metastatic status, none of the comorbidity indices showed a statistically significant association with the use of multimodal treatment. Increasing age was independently associated with a lower likelihood of multimodal treatment, whereas metastatic status showed no significant effect.

Similar results were observed for the relationship between comorbidity scores and the prescribed EQD2 levels. Patients with lower cCCI and uCCI scores tended to receive higher cumulative EQD2 doses to the primary tumor, with statistically significant positive associations in univariable analyses (both *p* < 0.01). In contrast, aaCCI showed no meaningful association with EQD2 levels, and no clear correlation was observed for the SCS. (see Table 6).

**Table 6:** Correlation between comorbidity score and applied radiotherapy dose (EQD2)

Comorbidity score	EQD2 level	
	Spearman correlation coefficient (ρ)	Significance (*p*)
cCCI	−0.39	**< 0.001**
uCCI	−0.40	**< 0.001**
aaCCI	−0.07	**0.047**
SCS	0.20	**0.04**

Bold figures indicate statistical significance (*p* < 0.05)

EQD2, Equivalent dose in 2 Gy fractions; cCCI, conventional Charlson Comorbidity Score; uCCI, updated Charlson Comorbidity Score; aaCCI, age-adapted Charlson Comorbidity Score; SCS, Simplified Comorbidity Score

In multivariable models adjusted for age and metastatic status, none of the comorbidity indices (*p* > 0.05) but KPS (*p* < 0.001) were independently associated with the applied EQD2 dose levels.

## Discussion

To the best of our knowledge, this is the first comprehensive report investigating comorbidities as a potential prognostic factor for OS and the influence on treatment allocation in patients with ATC. In our analysis, the cCCI demonstrated moderate discriminatory ability for predicting 6-month survival, with the highest AUC among the evaluated comorbidity scores. Although its odds ratios and reclassification metrics (NRI, IDI) suggest some prognostic contribution, the overall effect remained limited. The uCCI showed comparable but slightly weaker performance and may serve as an alternative when detailed clinical information for cCCI calculation is unavailable. We also noted that higher comorbidity burden (cCCI and uCCI) correlated with lower treatment intensity. However, after adjusting for relevant clinical cofactors such as age and metastatic status, neither comorbidity score retained independent prognostic significance. In contrast, established prognostic markers particularly performance status measured by KPS remained as a strong and clinically meaningful predictor of survival.

In the past, several prognostic factors were found to have strong impact on survival in ATC. These include UICC stage, occurrence of metastases, treatment modality and applied radiation doses (Haymart et al. 2013; Fan et al. 2019; Glaser et al. 2016; Wendler et al. 2016). However, number and severity of comorbid diseases have not yet been taken into consideration when predicting a patient’s outcome. National and international guidelines do not comment on comorbidities as potential prognostic factor in ATC patients due to a lack of evidence (Filetti et al. 2019; Haddad et al. 2022), although the burden of comorbidities is gaining relevance since life expectancies and thus the incidence of cancer and other chronic diseases are constantly rising (Yancik et al. 1997). In addition, ATC occurs most often in elderly people (age > 65 years). To help physicians estimate a patients’ outcome based on different prognostic factors, an objective evaluation of comorbidities and their influence on a patients’ prognosis should always be included. Therefore, the availability and application of universally recognized and validated scoring systems, like the Charlson Comorbidity Index and its adaptions are crucial (Charlson et al. 1987). In several cancer entities, higher comorbidity burden was associated with lower OS, but data for ATC is lacking (Göllnitz et al. 2016; Robbins et al. 2013; Søgaard et al. 2013; Piccirillo et al. 2004; Rieker et al. 2002; Yamano et al. 2017). Maniakas et al. investigated the role of comorbidities using the age-adjusted Charlson comorbidity index (0–2 vs. 3–4 vs. ≥5) (Maniakas et al. 2020). Along with the results of Maniakas et al., we found that the aaCCI is not an independent prognostic factor for OS. In contrast to their findings, we identified the cCCI and the uCCI with a cutoff value of 2 as a prognostic factor for OS (*p* < 0.001). The recent NCDB analysis of Alhayek et al. found that a higher Charlson comorbidity score was consistently associated with worse OS and remained significance in multivariate analysis (uCCI score 1 vs. 0: HR: 1.189; 95% CI 1.088–1.299; *P* < 0.001; score 2 vs. 0: HR: 1.515; 95% CI 1.287–1.784; *P* < 0.001). Compared with patients with a score of 0, those with scores of 1 or 2 showed significantly increased mortality risks in the overall cohort, as well as in both metastatic and non-metastatic subgroups highlighting comorbidity burden as a strong and independent prognostic factor for OS in ATC.

To assess the impact of comorbidity burden on prognosis other confounding factors such as tumor stage and performance status must be taken into consideration. In a study by Lee et al. with a cohort of 2070 patients with differentiated thyroid carcinoma (DTC), patients with higher T, N and M classifications were more likely to die from cancer itself rather than comorbidities. A high number of comorbidities (≥ 3) however was associated with a very low probability of dying from DTC as patients rather died earlier from other causes, like non-thyroid malignancies or cardiovascular diseases (Lee et al. 2019). Another analysis by Edwards et al. confirms that age and comorbidity have a smaller impact on OS for patients with distant disease (Edwards et al. 2014). Because of rapid local progression and early metastasis, more than 50% of the patients with ATC already have a distant disease (stage IVC) at initial diagnosis and compared to DTC, ATC patients experience a dismal prognosis (Augustin et al. 2020). In multivariate analysis for OS, we found only a trend for KPS (*p* = 0.06) and type of therapy (*p* = 0.087) highlighting the prognostic value of classical prognostic factors and multimodal treatment.

For ATC, recent studies found multimodal treatment associated with improved OS (Pezzi et al. 2017; Haymart et al. 2013; Corrigan et al. 2019; Wendler et al. 2016; Prasongsook et al. 2017). As a consequence, national and international guideline recommendations implemented multimodal therapy as standard of care for patients with localized (stage IVA) and resectable regional (stage IVB) anaplastic thyroid cancer. ATC patients without genetic mutations show a dismal prognosis and experience severe treatment-related toxicity/complications of conventional treatment (Bible et al. 2021; Filetti et al. 2019). Consequently, all ATC patients should receive molecular profiling to detect potential druggable mutations (Haddad et al. 2022; Wang et al. 2022). Targeting BRAF-V600E mutations with a combination of Dabrafenib and Trametinib has shown promising results and was approved by the Food and Drug Administration (FDA) as a first-line therapy for advanced or metastatic anaplastic thyroid cancer (Subbiah et al. 2018; Zhao et al. 2023). An alternative approach for non-druggable mutations is targeting the tumor’s microenvironment with immunotherapy such as Programmed-Death-Ligand-1(PDL-1) Inhibitors in combination with a multiple kinase inhibitor against the VEGFR1, VEGFR2 and VEGFR3 kinases. A prospective phase II trial by Dierks et al. evaluated 27 ATC patients with ATC undergoing lenvatinib plus pembrolizumab resulting in a partial response within 2 years of 51.9% and stable disease in 48.1% of patients (Dierks et al. 2022).

Local radiotherapy to the primary tumor can be offered to provide local and symptom control (Bible et al. 2021; Filetti et al. 2019). The prescribed cumulative radiation dose depends on the intention of treatment (curative vs. palliative), setting (definitive vs. adjuvant radiotherapy), patients’ performance status and extent of disease (localized vs. metastatic) (Bible et al. 2021). However, an analysis of data of the National Cancer Database (NCBD) found that ATC patients receiving higher (60–75 Gy) versus lower (45–59.9 Gy) therapeutic doses resulted in improved OS (Pezzi et al. 2017). In our study, we found that comorbidity burden is associated with treatment allocation and OS suggesting higher scores within the cCCI and uCCI significantly led to less aggressive treatment. This does not only concern treatment options, but also the level of cumulative radiation doses to the primary tumor. ATC patients with higher comorbidity burden in the cCCI and uCCI were irradiated with lower radiation doses, which may result in lower tumor control and worse OS. However, after adjusting for age and metastatic disease, none of the comorbidity indices (*p* > 0.05) but KPS (*p* < 0.001) were independently associated with the applied EQD2 dose levels. On the other hand, ATC patients with a high comorbidity score (≥ 2 points) in the cCCI have a median survival of only 1.8 months and may benefit more from best supportive care. For these patients, aggressive treatment approaches should be avoided, as they are likely to cause more harm than benefit, such as increased treatment-related toxicity, prolonged hospitalization and a reduced quality of life.

Our study found that comorbidity burden has a clinically relevant impact on prognosis of patients with ATC, but should be interpreted in conjunction with performance status to ensure a more accurate prediction of OS. In fact, cCCI can help predicting patient’s outcome and assessment should be recommended before treatment planning. Moreover, comorbidity burden measured by cCCI and uCCI seem to influence physicians’ therapeutic decision-making. Patients with lower scores often receive more aggressive treatment and higher radiation doses and might therefore have a better outcome (local control and OS). On the other hand, assessing a patients comorbidity score might help to differentiate whether patients with a high comorbidity burden might also benefit from more aggressive treatment or to avoid intensive therapy maintaining quality of life and circumvent treatment-related toxicity.

Several limitations need to be considered such as the retrospective character of our multicenter study and the risk of hidden selection biases. KPS data in our cohort was missing in a relevant proportion (32.8%), but remained a relevant prognostic factor for OS. The lack of KPS data represents a relevant limitation that may have affected effect estimates. Furthermore, NGS was only performed in 21.9% of all patients. It is important to note that the data was collected over an extended period, during which advancements in targeting druggable mutations—particularly in elderly patients—may have mitigated the impact of comorbidities on prognosis, thereby weakening the observed correlation. In addition, detailed information on metastatic sites was not uniformly available across participating centers and was therefore not included in our analysis. Overall, these factors highlight the need for prospective, systematically collected patient registry to validate our findings and further clarify the interplay between comorbidities, performance status, and treatment outcomes in ATC.

In summary, patients’ comorbidities along with performance status are prognostic factors for OS in ATC patients and should be taken into consideration for treatment allocation. The conventional Charlson Comorbidity Score was particularly effective compared to other comorbidity scores in predicting patient’s prognosis at 6 months after radiotherapy.

## Supplementary Information

Below is the link to the electronic supplementary material.

Supplementary Material 1

Supplementary Material 2

## Data Availability

Data are available from the corresponding author upon reasonable request.
