# Supplementary material for: Impact of comorbidities on treatment management and prognosis in patients with anaplastic thyroid cancer (ATC)
Source: J Cancer Res Clin Oncol. 2025 Dec 23;152(1):22. doi: 10.1007/s00432-025-06403-7 (PMC12728151; doi:10.1007/s00432-025-06403-7)
Supplement: Supplementary file 1 — Supplementary Material 1 [file 432_2025_6403_MOESM1_ESM.docx]

**Supplementary Table SI:** Conventional and Updated Charlson Comorbidity Index

| **Comorbid conditions** | **cCCI** | **uCCI** |
| --- | --- | --- |
| Myocardial Infarction | 1 | 0 |
| Congestive Heart Failure | 1 | 2 |
| Peripheral Vascular Disease | 1 | 0 |
| Cerebrovascular Disease | 1 | 0 |
| Dementia | 1 | 2 |
| Chronic Pulmonary Disease | 1 | 1 |
| Rheumatic Disease | 1 | 1 |
| Peptic Ulcer Disease | 1 | 0 |
| Mild liver Disease | 1 | 2 |
| Diabetes without chronic complication | 1 | 0 |
| Diabetes with chronic complication | 2 | 1 |
| Hemiplegia or Paraplegia | 2 | 2 |
| Renal Disease | 2 | 1 |
| Any malignancy without metastasis | 2 | 2 |
| Leukemia | 2 |  |
| Lymphoma | 2 |  |
| Moderate or Severe Liver Disease | 3 | 4 |
| Metastatic Solid Tumor | 6 | 6 |
| AIDS (excluded asymptomatic infection) | 6 | 4 |
| **Maximum comorbidity score** | **33** | **24** |
