# Supplementary material for: Impact of comorbidities on treatment management and prognosis in patients with anaplastic thyroid cancer (ATC)
Source: J Cancer Res Clin Oncol. 2025 Dec 23;152(1):22. doi: 10.1007/s00432-025-06403-7 (PMC12728151; doi:10.1007/s00432-025-06403-7)
Supplement: Supplementary file 2 — Supplementary Material 2 [file 432_2025_6403_MOESM2_ESM.docx]

**Supplementary Table SII:** Simplified Comorbidity Score (SCS)

| **Comorbid conditions** | **Points** |
| --- | --- |
| Tobacco consumption | 7 |
| Diabetes mellitus | 5 |
| Renal Insufficiency | 4 |
| Respiratory Comorbidity | 1 |
| Cardiovascular Comorbidity | 1 |
| Neoplastic Disease | 1 |
| Alcoholism | 1 |
